# Effect of Hearing Aids on Phonation and Perceived Voice Qualities

**DOI:** 10.1177/23312165251322064

**Published:** 2025-03-03

**Authors:** Johanna Hengen, Inger Lundeborg Hammarström, Stefan Stenfelt

**Affiliations:** 1 4566 Department of Biomedical and Clinical Sciences, Linköping University, Linköping, Sweden

**Keywords:** hearing loss, hearing aids, phonation, acoustics

## Abstract

Problems with own-voice sounds are common in hearing aid users. As auditory feedback is used to regulate the voice, it is possible that hearing aid use affects phonation. The aim of this paper is to compare hearing aid users’ perception of their own voice with and without hearing aids and any effect on phonation. Eighty-five first-time and 85 experienced hearing aid users together with a control group of 70 completed evaluations of their own recorded and live voice in addition to two external voices. The participants’ voice recordings were used for acoustic analysis. The results showed moderate to severe own-voice problems (OVP) in 17.6% of first-time users and 18.8% of experienced users. Hearing condition was a significant predictor of the perception of pitch in external voices and of monotony, lower naturalness, and lower pleasantness in their own live voice. The groups with hearing impairment had a higher mean fundamental frequency (f0) than the control group. Hearing aids decreased the speaking sound pressure level by 2 dB on average. Moreover, acoustic analysis shows a complex relationship between hearing impairment, hearing aids, and phonation and an immediate decrease in speech level when using hearing aids. Our findings support previous literature regarding auditory feedback and voice regulation. The results should motivate clinicians in hearing and voice care to routinely take hearing functions into account when assessing voice problems.

## Introduction

Previous studies have investigated the difference in perceived voice problems among hearing aid users, individuals with unaided hearing impairment, and individuals with age-appropriate hearing (Coelho, Brasolotto, et al., 2015; Hengen et al., 2018, 2020; Laugesen et al., 2011). While these studies identified significant differences in self-rated voice problems and descriptions of voice qualities across these groups, the current study aims to investigate the immediate impact of hearing aid use on self-perceived voice. Specifically, it seeks to determine whether hearing aids have an instant effect on the perception of one's own voice and if altered auditory feedback influences measurable voice properties. Understanding whether the perception of voices and phonation changes with hearing aid use is crucial for both audiologists and speech-language pathologists. Addressing this problem is important because disuse of hearing aids remains relatively common (Franks & Timmer, 2023), and voice therapy may be complicated by potentially disturbed auditory feedback of one's own voice. The rationale for this hypothesis is outlined below.

The sound of a voice carries information regarding the gender, age, and cultural identity of the speaker. The nuances in our voices convey emotional states (Banse & Scherer, 1996; Scherer et al., 2001), and can change depending on who we are talking to (Bailly & Martin, 2014; Leongómez et al., 2017).

Research has shown that people tend to rate their own voice as more attractive than others, referred to as vocal implicit egotism (Hughes & Harrison, 2013). Most people are likely to agree that they, to an extent, have a special relationship to the sound of their own voice compared to the voices of others. When a person perceives their own voice to have changed, they may feel like it no longer expresses identity. For example, the voices of individuals with Parkinson's disease typically become more monotone as the condition progresses (Kent & Rosenbek, 1982). Monotony is usually interpreted by listeners as coming from an emotionless speaker (Pell et al., 2006). The term “voice dysphoria” is mostly used in the transgender community, but the concept is applicable to other circumstances where the individual does not feel that their voice aligns with their identity.

Due to the technical design of hearing aids, it is currently unavoidable that the acoustic signal of the own voice (and the voices of others) change to a degree, although this does not necessarily mean that the change is negatively perceived by the hearing aid user. Considering the uniqueness of a speaker's voice, a change in own-voice sound quality may evoke a reaction in hearing aid users which is different from a change in general sound quality. For most users, one can hope that this is a positive change, with a clearer signal that allows users to understand other people's speech better and perceive tonal details that they otherwise would not have heard. However, displeasure with the sound of the own voice is a relatively common occurrence in hearing aid users (Kochkin, 2002, 2005, 2010).

The way we experience our own voice does to a degree affect how we use it (Keough et al., 2013; Lane & Tranel, 1971). In 1911, French otorhinolaryngologist Étienne Lombard published an article detailing an observation that a patient's vocal amplitude increased when presented with an intense sound (Lombard, 1911). This discovery founded the phenomenon which is now referred to as the Lombard effect—an involuntary vocal adaptation in response to altered auditory feedback. Previous research has examined the function of the auditory system during phonation by altering the auditory environment and observing vocal response (e.g., Lane & Tranel, 1971; Purcell & Munhall, 2006a, 2006b; Villacorta et al., 2007; Xu et al., 2004).

The effect of auditory regulation on phonation is probably best demonstrated by studies exploring the voices of individuals with profound hearing loss with onset in childhood, which have found deviations ranging from smaller prosodic differences to severe vocal dysfunction (Coelho, Medved, et al., 2015). The large variation is likely due to differences in methodology between studies and the heterogeneous nature of the samples (hearing loss etiology and severity, use of hearing aids or cochlear implants, pre- or postlingual onset), according to a review of the area (Coelho, Medved, et al., 2015). Few voice-related studies have been conducted on elderly individuals with acquired hearing impairments. This is problematic as the prevalence of hearing impairments in this group is high (Mattos & Veras, 2007).

One study from 2012 with only males found significantly higher f0 values among 30 participants with sensorineural hearing impairment (age range 35–53 years) compared to 30 participants with normal hearing (age range 38–51 years). On average, the speech f0 of males with hearing impairment was +17 Hz higher than that of the normal-hearing individuals (Mora et al., 2012). A cross-sectional descriptive study from 2007 found increasing values of f0 in 30 elderly women with hearing impairment related to gradually sloping audiometric curves (Baraldi Gdos et al., 2007).

Since hearing impairment and hearing aids both alter the auditory feedback to the speaker, either could theoretically cause vocal changes. The question of whether hearing aids specifically have a significant impact on phonation is largely unknown. In a previous study (Hengen et al., 2018), hearing aid users were found to have a higher score on the Voice Handicap Index compared to individuals with unaided hearing loss and a control group with age-appropriate hearing. However, there were some confounding factors, as the hearing aid users had a higher average age than the two other groups (Hengen et al., 2018). It is also unknown whether hearing aids have an immediate effect on phonation or if potential vocal changes occur gradually over a longer period of time.

If hearing aids influence phonation, the cause lies in the auditory feedback or the altered perception of the auditory environment. The personal experience of the own voice is an important aspect to consider in this type of research. Furthermore, it is highly relevant to understand what hearing aid users hear when they complain of own-voice problems (OVP), both during potential voice counseling and hearing aid fitting. According to a survey from 2003, OVP were one of the most common issues audiologists were faced with when fitting patients with hearing aids (Jenstad et al., 2003). In two previous studies, the percentage of hearing aid users who were satisfied with the sound of their own voice ranged from 41% to 73%, an indisputably large variation (Høydal, 2017; Kochkin, 2010). In the past, OVP in hearing aid users were mostly attributed to the occlusion effect (Dillon, 2012).

The occlusion effect occurs when an object (such as a hearing aid mold) occludes the ear canal. Bodily generated sounds (such as the voice) are transmitted through both air- and bone (and other bodily tissues) pathways (Stenfelt & Goode, 2005; Stenfelt, 2020). These pathways are referred to as air-conducted (AC) and bone-conducted (BC) and contribute approximately equally to the speaker's perception of their own voice (Reinfeldt et al., 2010). At low frequencies, the radiation impedance of the open ear canal and the BC induced ear canal sound pressure are low (Stenfelt et al., 2003; Surendran & Stenfelt, 2022). In the case of an occluded ear canal, the sound pressure of these sounds increases due to a higher termination impedance (Dillon, 2012; Stenfelt & Reinfeldt, 2007). Hearing aid users experiencing the occlusion effect usually describe their voice as sounding “like talking in a barrel,” that is, boomy and hollow (Winkler et al., 2016). While the occlusion-related changes in the user's voice are only perceivable to the users themselves, it can be a very unpleasant experience to the user and lead to disuse of hearing aids (Dillon, 2012).

Occlusion-related problems have in modern times been reduced due to the increase of hearing aids with open fittings (dome inserts) and molds with increased ventilation canals (Winkler et al., 2016). The selection between a dome or a mold insert depends on the patients’ hearing needs and preferences (Dillon, 2012). Increasing the size of ventilation canals (to reduce occlusion) is weighed against the possibility for sufficient low-frequency amplification and feedback problems (Winkler et al., 2016). A recent study comparing six different earmold designs found that, consistent with previous research, the opening of the ear canal, as described by the acoustic mass of the vent, was the strongest predictor of the perceived occlusion effect in the subjects (Denk et al., 2023).

Less has been published regarding dissatisfaction of own-voice sounds in hearing aid users with domes. In the survey by Høydal (2017) of 384 participants, the majority had dome inserts and only 41% were satisfied with the sound of their own voice (Høydal, 2017). Laugesen et al. (2011) write in their conclusion “Overall, this study implies that while open fittings provides a tremendous reduction in the occlusion-related OVP for hearing-aid users, there is still progress to be made on other dimensions of own voice” (Laugesen et al., 2011). Even when the patient is satisfied with the sound of their own voice, there are several reasons to believe that the hearing aids alter its acoustic properties, although hearing aid users may not consciously perceive these changes.

Firstly, as mentioned above, the sound of the own voice consists of an approximately equal proportion of AC and BC sounds (Reinfeldt et al., 2010). Sensorineural hearing loss is characterized by higher hearing thresholds for both types of sounds, but normal-hearing aids only amplify AC sounds, which increases the contribution of AC sounds, relative to BC sounds (Stenfelt, 2011). The BC components of the voice are believed to be the main reason why people often perceive their own voice to sound different when hearing it during live phonation (deeper, fuller), compared to when played back from a recording with only AC sounds (Hullar, 2009). Another possible reason for the difference between the live and recorded own voice is the spatial filtering effect, where sound in front of the mouth, where a recording microphone is often placed, has different spectral content than the own voice recorded at the ear (Cornelisse et al., 1991). Even so, an alteration of the AC/BC balance could result in a noticeable difference in voice quality perception.

Further reasons for why the own voice may sound different during hearing aid use include the slight time delays in hearing aids, which leads to a minor time difference between the AC- and BC components (Stone & Moore, 1999). However, this is unlikely to be noticeable to most hearing aid users. The time delay also affects spectral content of the AC signal with open fittings, due to the comb-filter effect. Such comb-filtering affects both the own-voice signal and external signals. The effect of delay on the comb-filter is most noticeable for hearing aid users with mild hearing losses (Lelic et al., 2022). Dynamic range compression, directional microphones, sound processing, and individual settings in the hearing aids can also alter how the own voice is perceived (Dillon, 2012; Kuk & Ludvigsen, 2002; Stenfelt, 2011). It is also worth considering that hearing loss affects the tonal quality of all sounds, which means that a first-time (FT) user of hearing aids may need some time to acclimatize when a hearing aid restores it to a closer approximation of their original voice quality. However, this is deemed an unlikely reason for OVP (Dillon, 2012).

There are few previous studies that elaborate on the experienced own-voice quality among hearing aid users. Laugesen et al. (2011) used a self-developed questionnaire for investigating OVP in hearing aid users. The results of this study indicated that hearing aid users had more problems regulating vocal sound pressure level (SPL) and speaking and hearing at the same time, compared to a control group with age-appropriate hearing (Laugesen et al., 2011). A previous publication by Hengen et al. (2020) attempted to separate the effects of the hearing impairment and the hearing aids on the own-voice perception, and found that hearing aids were more likely to affect sound quality issues and the ability to speak while simultaneously listening. The same study found that own-voice dissatisfaction increased post-hearing aid fitting among FT users (Hengen et al., 2020). However, to the best of our knowledge, there are no studies that compare the perception of the own and external voices with or without hearing aids in a same-day, live, controlled setting.

This study aimed to investigate how hearing aids affect the perception of one's own voice and the voices of others in hearing aid users. It also sought to determine whether this altered perception could lead to immediate changes in vocal behavior, which could be identified through acoustic voice analysis. The hypothesis was that hearing aids alter the perception of both the user's own voice and other people's voices, and that this would be noticeable to some hearing aid users. Additionally, the study aimed to investigate potential factors related to the hearing aid and user characteristics to see if they influenced scores in the perceptual voice evaluation.

## Method

### Design

The study was approved by the Regional Ethical Review Board in Linköping, Sweden (No. 2014/335-31). The sample included in this study took part in a larger investigation of own-voice experiences during hearing aid fitting, which took place at Linköping University Hospital in Sweden. Some data from this larger investigation have already been published (Hengen et al., 2018, 2020). Participants were recruited into three groups: FT hearing aid users, experienced hearing aid users, and a control group without hearing impairment. Written informed consent was acquired from all the participants. More information regarding the recruitment can be found in the publications by Hengen et al. (2018, 2020). None of the participants received compensation for their participation in the study.

### Participants

The main inclusion criteria were that the FT users had no experience with hearing aids, and that the experienced users had a minimum of 2 years’ experience with hearing aids at the time of refitting. The control group had to pass a hearing test before inclusion in the study. Hearing thresholds up to 25 dB HL at and below 1 kHz, 30 dB HL at 2 kHz, 40 dB HL at 4 kHz, and 50 dB HL at 6 kHz, were allowed, as it was deemed likely to exclude any individuals who would benefit from a hearing aid from the control group. This limit was also used in Laugesen et al. (2011) with the same rationale. The sample included 85 participants each in the FT user (54 male, 31 female) and experienced user groups (52 male, 33 female). The control group consisted of 70 participants (33 male, 37 female). The sample sizes are different compared to Hengen et al. (2018, 2020) as the previous studies excluded participants who did not submit the questionnaires used for those studies. The current study included everyone who participated in the onsite tests. The age of the participants was 70 ± 10 years old (mean ± *SD*) for the FT users, 75 ± 9 years old for experienced users, and 70 ± 9 years old for the control group participants. The pure-tone average (PTA) for the better ear was 13 ± 10 dB for the control group, 31 ± 10 dB for the FT users, and 45 ± 16 dB for the experienced users.

The control group were tested once and underwent a shorter investigation, as the tests were only conducted without hearing aids. The two patient groups participated in two sessions, one at the beginning and one at the end of their hearing aid fitting. The hearing aid fitting process was different for each patient, but the average time between the first and the final visit was approximately 4 months for the FT users (122 ± 68 days) and 3.5 months (110 ± 78 days) for the experienced users. The fitting process consisted of an average of 4 ± 1 visits for the FT users and 4 ± 2 visits for the experienced users. The experienced hearing aid users underwent hearing aid refitting due to the current devices needing to be replaced either due to age of the devices, malfunction, or changed hearing needs.

Hearing aid fitting was performed by trained hearing care specialists at the clinic. The study did not interfere with the fitting process; decisions regarding hearing aid model, type of inserts, and settings were made collaboratively by the clinician and the patient. Medical notes from the fitting process were collected, but the analysis in this study was limited to PTA and the type of inserts used. This study can be described as a non-interventional clinical study.

The fitting rules followed were those recommended by the manufacturer for their hearing aids. If the initial fitting was unsatisfactory for the patient, a NAL-NL2 (Keidser et al., 2011) fitting was employed. A real-ear measurement (REM) of the hearing aid gain was conducted at the final visit, not as a fitting target but as a verification to ensure the gain was reasonable and free of spurious spectral components. Behind-the-ear hearing aids were the most common fittings (*n* = 60 for FT users, *n* = 56 for experienced users).

Table 1 provides a summary of the fitted hearing aids, categorized by hearing aid user groups and whether the fittings were monaural or bilateral. Domes were more common among FT users (*n* = 64) compared to molds (*n* = 21), whereas molds were more prevalent in the experienced user group (*n* = 56 compared to *n* = 29 for domes).

**Table 1.** Summary of Hearing Aids Fitted for Participants in the Two Hearing Aid User Groups.

		First-time users		Experienced users	
Manufacturer	Model	Mono	Bilateral	Mono	Bilateral
Beltone	Boost			1	
	Silk	1	5		9
Oticon	Alta		2	1	5
	K220	1	8	2	12
Phonak	Ambra		1		5
	Audeo			1	
	Bolero	3	13	1	12
	Exelia				1
	Virto		1		1
	Versata			1	1
Siemens	Carat		3		
	Motion	2	10		8
	Pure	1	10		5
Widex	Clear		4		4
	Dream	5	14	2	11
	Mind			1	

### Data Collection

The data collection consisted of recordings and voice evaluations. The audio recordings were conducted in a sound-attenuated booth at the clinic. Participants were positioned 20–30 cm in front of a microphone (Brüel & Kjær type 4189) with a foam pop filter and recorded using the Brüel & Kjær Pulse 18.0 system. The reading material was displayed on a screen with a large font in front of the participants. Every participant was asked before the recording if the text size was sufficient. Another version of the text with a bigger font was used for individuals with vision impairment. The participants were asked to read the short story “The north wind and the sun.” The participants in the control group were recorded once, while the participants in the patient groups were recorded three times: before hearing aid fitting and refitting (unaided) and at the conclusion of hearing aid fitting (unaided and with hearing aids).

The acoustic analysis was conducted on a 10 s voice sample from the recording, using the software Praat (Boersma & Weenink 2017). The measurements collected included mean, minimum, and maximum fundamental frequency (f0); mean, minimum, and maximum SPL; jitter; shimmer; and mean harmonics-to-noise ratio (HNR). In Praat, SPL is computed as the root-mean-square amplitude over 0.1 s time windows and averaged over the 10 s voice sample to yield the mean SPL. In this study, SPL values were calculated using A-weighting. The choice of acoustic parameters was based on previous publications (such as Coelho, Medved, et al., 2015) on acoustic descriptions of voice qualities and was intended to reflect voice stability and vocal health.

The voice evaluations were completed in the same booth as the recordings took place in. In this test, the participants were asked to rate four different voice stimuli on a short questionnaire developed by the authors. At the conclusion of hearing aid fitting, the voice evaluation was repeated twice (with and without hearing aids). The order of the unaided and aided evaluation was balanced to average out procedural effects, meaning that half the participants completed the aided evaluation first, and the other half completed the unaided evaluation first.

The four voice stimuli evaluated were (a) the participant's own voice during live speech, (b) the recorded participant's own voice played back to them through a loudspeaker, (c) an unfamiliar voice, and (d) a familiar voice. The familiar voice was selected at the start of testing, where participants were asked how familiar they were with four well-known Swedish TV news anchors and radio speakers (two female and two male, ranging from middle-aged to late 60s). Recorded voices were used as they are only transmitted as AC sound. A familiar and unfamiliar voice was selected to investigate whether prior knowledge of how the voice is “supposed to sound” affected the ratings. A comparison of the own live voice and the own recorded voice was used to compare whether the effect of the sound source contributed to potential changes.

The recordings were played back to the participants in a sound-attenuated booth using an audiometer (Interacoustics AC 40, www.interacoustics.com) connected to a power amplifier (Rotel RB-03, www.rotel.com) and a loudspeaker (Tannoy system 800, www.tannoy.com). The sound field was evaluated by recording a 30 s band-limited white noise that was played through the system by a sound level analyzer (Brüel and Kjær 2250) equipped with a ½ inch microphone (Brüel and Kjær 4189). The sound levels were within 6 dB when analyzed in one-third octave bands from 100 Hz to 12.5 kHz. The equalization of the different speech materials was achieved by adjusting the level on the VU meter of the audiometer.

The unfamiliar voice samples were collected from four different individuals (one younger and one older female, one younger and one older male) located in a different city (and therefore unlikely to have ever interacted or been heard by the study participants before). The external voices were recorded in different locations due to practical limitations, but each recording took place in a quiet studio room with the same equipment to ensure similar recording conditions. The individuals who volunteered with the unfamiliar voice samples were not trained speakers, but they did not have any history of voice disorders and were considered to have an average or typical overall voice quality. The unfamiliar voice was selected by the test administrator with the intent to approximately match the voice stimuli to the participant's age and gender. The test administrator documented which participant had listened to which voices at the sessions to ensure consistency.

All the voice stimuli consisted of a reading of “the northwind and the sun.” The voices were not intended to be completely equal in terms of pitch or other voice qualities, and no statistical comparisons of between-voices ratings were planned.

As perceptual voice evaluations are generally done by trained speech-language pathologists or other expert listeners, the evaluation form had to be designed with non-experts in mind. The selection of voice parameters was based on two conditions; that the terms were likely to be understood by non-expert listeners and that they were relevant based on the theoretical background of how hearing aids may affect the perception of a voice. A short questionnaire was also considered preferable to avoid test fatigue, so only six parameters were selected. As with the other materials used in this study, the voice evaluation questionnaire was written in Swedish and therefore some terms do not have an exact translation, but the best English matches for the parameters used are “pitch,” “strain,” “hoarseness,” “prosody/monotony,” “naturalness/unnaturalness,” and “pleasantness/unpleasantness.” The response scale was 0–8, with opposite terms at each end of the scale. On the “pitch” parameter, 0 was “very low pitch” and 8 was “very high pitch.” The rest of the parameters were designed so that the presence of “negative” qualities, such as “hoarseness,” a high score would indicate severe hoarseness, and a low score *none* (0) or *very little* (2–3) hoarseness. The participants were asked if they understood the tasks and if they had any questions before the ratings began.

The participants were encouraged to ask the test administrator to replay the recordings if needed. During the listening, the participants could ask for the volume to be set to a comfortable level where the voice was clearly audible but not too loud. The requests were done using predetermined hand signals. The volume adjustments were not documented, and subjects could ask for the volume to be increased or decreased during the task. The initial setting was 60 dB SPL.

Data will be made available upon reasonable request.

### Analysis

As this study consisted of different types of data, several different statistical analyses were used. The data from the acoustic analysis passed the assumptions for parametric tests and were therefore examined with analysis of variance (ANOVA) and *t*-tests with Cohen's *d* for effect size (for comparisons of equal samples) and Hedges’ correction (for comparisons of unequal samples).

As the repeated voice evaluations were on an ordinal level, this required a non-parametric approach. Generalized estimating equations (GEE) were selected due to their ability to handle repeated measurements on an ordinal scale with a variation of different correlation data structures. A limitation of GEE is that it requires an approximately equal number of iterations for each case. As the control group only participated at the first timepoint, they were excluded from the GEE analysis. For the same reasons, the patients’ scores from the first session could not be included in the analysis, as it was only done for the unaided condition and not for the aided condition. However, descriptive data regarding both the control groups’ and the hearing aid user groups’ scores from all evaluations can be found in attachment 1. A robust variance estimator was used to account for the uncertainty about the correlation structure. As the selected model was ordinal logistic (due to the dependent variable being ordinal), this rules out Goodness of Fit scores. Significant factors in the GEE model are therefore only described by *p*-value, beta (*B*), and Wald chi-square (χ^2^). The exponentiated beta values are interpreted as *OR*. Insignificant results are only described with *p*-values.

The acoustic results were analyzed by two methods. The unaided baseline values (before hearing aid fitting) were compared using one-way ANOVAs and independent *t*-tests. A repeated measures mixed-model approach was used to predict the effect of hearing aids on phonation based on the values from the second session. The dependent variables consisted of the corresponding acoustic values and the between-subject factors were group, gender, and OVP, with age as a continuous predictor.

## Results

### Demography of OVP

Descriptive statistics were used to examine whether the participants with OVP differed in characteristics compared to the ones without OVP. The scores on the Own Voice Qualities questionnaire—item I1 “The sound of my own voice is a problem for me” were recoded into a binary variable. Our earlier publication used a broader inclusion of OVP, which categorized users as having “some OVP” if they scored over 2 on the 0–8 scale of agreement (Hengen et al., 2020). The current study uses a stricter inclusion criterion, where a score of 4 or higher is categorized as having OVP. These participants are therefore deemed as having moderate or severe OVP, compared to the previous study where a mild degree of OVP was accepted (Hengen et al., 2020).

**Figure 1.**
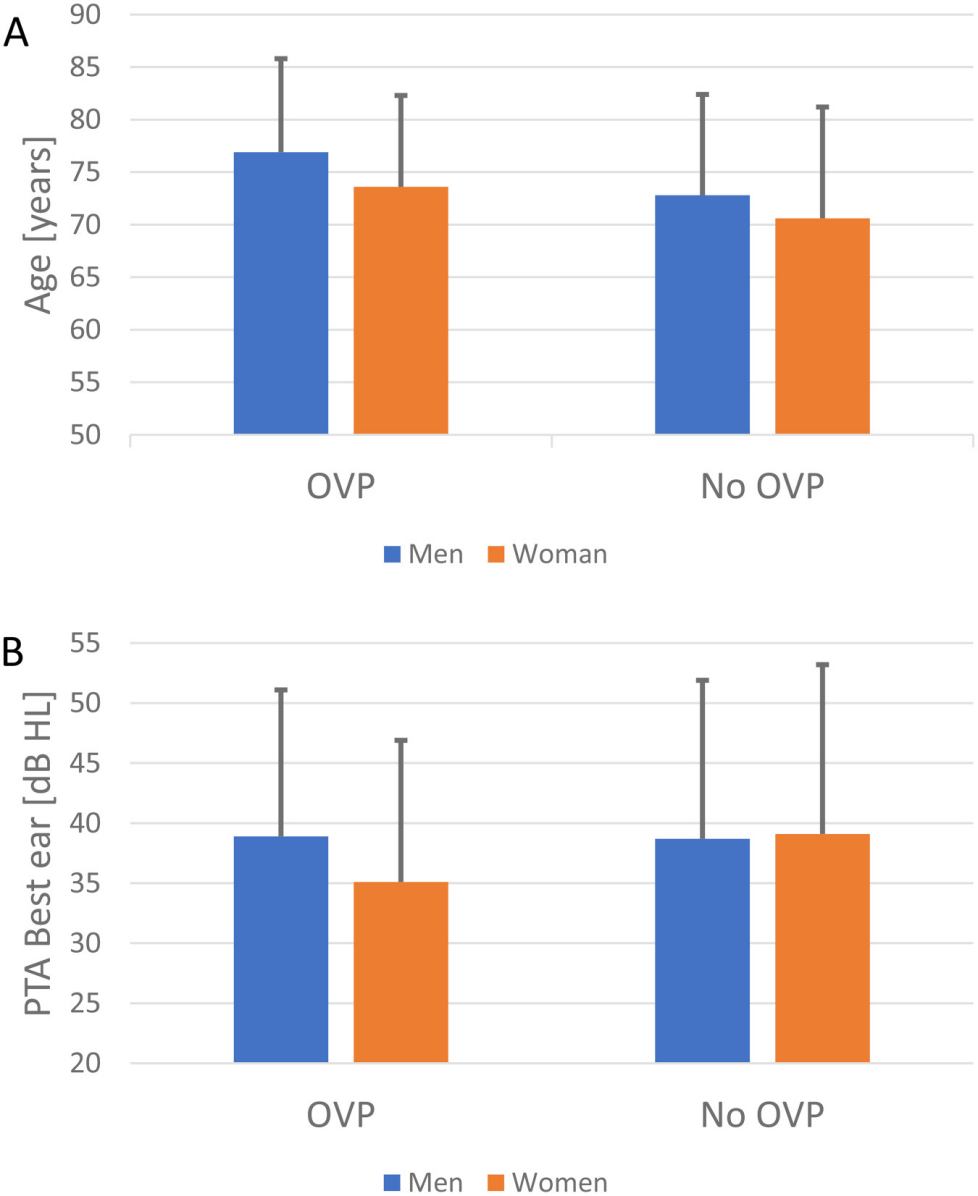
(A) Age and (B) PTA of the best ear for the men and women in the OVP and No-OVP groups.

*Note.* PTA = pure-tone average; OVP = own-voice problems.

The division resulted in 17.6% (*n* = 15) FT users and 18.8% (*n* = 16) experienced users in the OVP group. The (relatively) small number of hearing aid users reporting OVP according to this division limits the choice of statistical analyses and their validity. The data regarding the demography of this group are therefore presented only with descriptive statistics. The proportion of FT/experienced users, hearing aid inserts, male/females, and bilateral/unilateral fittings can be viewed in Figure 1.

**Figure 2.**
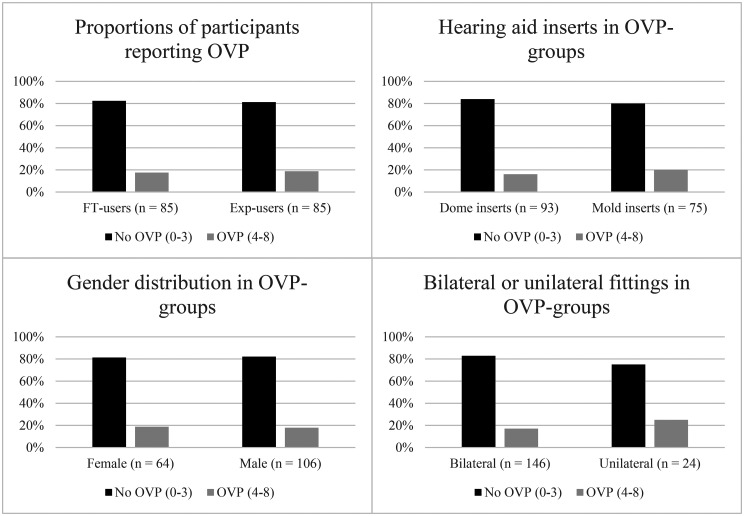
Demography of participants in OVP groups.

*Note.* OVP = own-voice problems.

At the conclusion of hearing aid fitting, there was no evident gender difference in terms of reporting OVP (see Figure 1). The proportion of users reporting OVP in relation to the total in the subgroup was higher for the unilaterally fitted users, where 25.0% of users reported having OVP, compared to 17.1% of the bilaterally fitted users. However, the unilaterally fitted group in total only included 24 participants, so the findings could be due to chance. The average AC PTA for the groups with and without OVP can be seen in Figure 2. The PTA for both ears appeared approximately equal between the “No-OVP” and “OVP” groups. Men with OVP had a significantly higher age than those without, but no age differences were found among the women (see Figure 2).

### Voice Evaluation

An overview of the voice evaluation can be found in Table 2. Average ratings for the different voices are available in Supplementary Appendix 1. The analysis was conducted for the four types of voices separately, and the results were compared to see how well the same model explained the variance and whether the effect of the factors varied depending on the voice. A robust variance estimator was used to account for the uncertainty about the correlation structure and only main effects were selected for each factor. The analysis was repeated for each voice parameter. The factors included in the analysis were: group (FT users and experienced users), inserts (dome or mold), gender (female or male), and hearing condition (unaided or hearing aid).

**Table 2.** Simple Overview of Significant Factors in the GEE Models of Voice Evaluation Data

Voice stimuli	Pitch	Strain	Hoarseness	Monotony	Natural	Pleasant
Familiar	HC—HA↑	*ns*	HC—HA↓	*ns*	Inserts—mold↓	Inserts—molds↓ Gender—male↓
Unfamiliar	HC—HA↑		*ns*	*ns*	HC—HA↓	
Own (recorded)	HC—HA↑ Gender—male↓		*ns*	*ns*	HC—HA↓	HC—HA↓
Own (live)	Gender—male↓		*ns*	HC—HA↑	Inserts—mold↓	HC—HA↓ Inserts–molds↓

*Note.* Factors included in the model: group (FT and experienced users), inserts (dome/mold), gender, hearing condition (unaided or hearing aid). GEE = generalized estimating equations; HC = hearing conditions; HA = hearing aid; *ns* = nonsignificant; FT = first-time users.

#### Pitch

Hearing condition was a significant predictor in the models for the recorded own voice (*p* < .01, *B* = 0.433, Wald χ^2^ = 10.231, *OR* = 1.542), the unfamiliar voice (*p* < .001, *B* = 0.578, Wald χ^2^ = 14.389, *OR* = 1.783) and the familiar voice (*p* < .001, *B* = 0.393, Wald χ^2^ = 7.585, *OR* = 1.481). Using hearing aids during the voice evaluation appears to increase the perceived pitch for recorded but not for live voices.

Gender was a significant factor for the live own voice (*p* < .01, *B* = −0.603, Wald χ^2^ 6.519, *OR* = 0.548) and the recorded own voice (*p* < .05, *B* = 0.601, Wald χ^2^ = 5.711, *OR* = 0.549), indicating that male participants in both cases were more likely to pick a lower pitch. It was not a significant factor for either the familiar or unfamiliar voice. Group or hearing aid inserts was not a significant factor in any of the GEE models for pitch (*p* ≥ .05).

#### Strain

There were no significant factors affecting ratings of strain in the models for either of the voices (*p* > .05), indicating that none of the factors in the model were likely to predict a change of perceived strain.

#### Hoarseness

Hearing condition was a significant factor for ratings of hoarseness in the familiar voice (*p* < .05, *B* = −0.31, Wald χ^2^ = 4.923, *OR* = 0.733), indicating that the hearing aid decreased the perceived hoarseness. There were no significant factors affecting ratings of hoarseness in the models for live own voice, recorded own voice and the unfamiliar voice (*p* > .05).

#### Monotony/Prosody

Hearing condition was the only significant factor for the live own voice (*p* < .05, *B* = 0.394, Wald χ^2^ = 5.864, *OR* = 1.675), suggesting that the aided condition increased the perceived monotony in the user's own voice during speech. There were no significant factors affecting ratings of prosody (monotony) in the recorded own voice, the unfamiliar voice and the familiar voice (*p* > .05).

#### Natural

Hearing condition was a significant factor affecting ratings of naturalness in the recorded own voice (*p* < .05, *B* = 0.324, Wald χ^2^ = 4.826, *OR* = 1.323) and the unfamiliar voice (*p* < .05, *B* = 0.273, Wald χ^2^ = 4.301, *OR* = 1.314). This meant that hearing aids decreased the perceived naturalness for these voices.

Hearing aid inserts had a significant effect on the live own voice (*p* < .05, *B* = −0.511, Wald χ^2^ = 4.756, *OR* = 1.666) and the familiar voice (*p* < .05, *B* = 0.604, Wald χ^2^ = 5.688, *OR* = 1.829). Mold inserts were associated with a less natural quality in the live own voice and in the familiar voice. The remaining factors (group and gender) did not have a significant effect on naturalness in any of the models.

#### Pleasantness

Hearing condition had a significant effect on ratings of pleasantness for the live own voice (*p* < .01, *B* = 0.469, Wald χ^2^ = 8.659, *OR* = 1.598) and the recorded own voice (*p* < .05, *B* = 0.312, Wald χ^2^ = 4.636, *OR* = 1.366). The direction of the effect was negative, meaning that hearing aids increased the odds of perceiving live own voice and recorded own voice as being less pleasant.

Hearing aid inserts was a significant factor in the model for the live own voice (*p* < .05, *B* = 0.567, Wald χ^2^ = 5.112, *OR* = 1.763) and the familiar voice (*p* < .01, *B* = 0.516, Wald χ^2^ = 4.211, *OR* = 1.446). Mold inserts were associated with lower pleasantness. Male gender was associated with a higher score of pleasantness in the familiar voice. Group was not a significant factor in any of the models.

### Voice Evaluation and OVP

The authors hypothesized that users reporting OVP experienced a more pronounced alteration in auditory feedback of their own voice than participants who did not report OVP. To investigate this, a subgroup analysis was conducted to compare whether OVP participants experienced a greater perceptual shift in voice evaluation parameters, focusing particularly on their own-voice conditions but also considering the unfamiliar/familiar condition. The binary variable of OVP was used as the grouping variable for this analysis.

Wilcoxon signed-rank tests were employed separately for each group to compare ratings with and without hearing aids for each parameter and voice signal. For the own (live) voice, a Wilcoxon signed-rank test indicated that evaluation ranks for pitch were statistically significantly higher when the No-OVP group was using hearing aids, *z* = −2.994, *p* = .003. There was no significant difference for the same parameter and voice in the OVP group.

The OVP group had significantly higher evaluation ranks of monotony and lower naturalness and pleasantness when using hearing aids (*z* = −2.134, *p* = .033 for monotony; *z* = −1.984, *p* = .047 for natural and *z* = −2.610, *p* = .009 for pleasant). The other comparisons for the live own voice did not show statistically significant results.

The Wilcoxon signed-rank tests indicated that the recorded own (*z* = −3.2, *p* = .001 “No-OVP” and *z* = −2.151, *p* = .031 “OVP”), unfamiliar (*z* = −3.037, *p* = .002 “No-OVP” and *z* = −2.285, *p* = .022 “OVP”) and familiar (*z* = −2.561, *p* = .01 “No-OVP” and *z* = −2.170, *p* = .032) voice had significantly higher pitch ranks during hearing aid use, regardless of group. The remainder of the parameters were not significantly different.

### Acoustic Analysis: Baseline Measures

Due to known gender differences in voice qualities, the baseline analysis was conducted for males and females separately. A preliminary analysis compared groupwise differences between the six subgroups (3 user types × gender, Tables 3 and 4), which will be presented below. Significance values for the between-groups measurements were adjusted to *p* ≤ .0036. The adjustment was calculated as the original alpha value divided by the total number of comparisons per parameter (14).

**Table 3.** Mean, Minimum, and Maximum f0 (Means and Standard Deviations)

		Mean f0 (Hz)			Minimum f0 (Hz)			Maximum f0 (Hz)		
Group	Gender	T1 NHA	T2 NHA	T2 HA	T1 NHA	T2 NHA	T2 HA	T1 NHA	T2 NHA	T2 HA
FT users	Male	139 ± 17	138 ± 15	134 ± 15	79 ± 10	78 ± 9	76 ± 9	353 ±94	345 ± 88	365 ± 117
	Female	206 ± 23	197 ± 21	196 ± 20	89 ± 14	94 ± 22	98 ± 20	369 ± 101	364 ± 96	418 ± 124
Experienced users	Male	149 ± 19	143 ± 16	142 ± 16	85 ± 7	84 ± 9	85 ± 16	356 ± 100	344 ± 82	369 ± 117
	Female	199 ± 15	199 ± 16	196 ± 17	98 ± 16	96 ± 17	97 ± 19	397 ± 88	405 ± 53	417 ± 94
Control group	Male	126 ± 11			70 ± 6			344 ± 70		
	Female	175 ± 19			95 ± 19			368 ± 93		

*Note.* f0 = fundamental frequency; FT = first-time users; NHA = no hearing aid; HA = hearing aid.

**Table 4.** Mean, Minimum, and Maximum SPL Measurements (Means and Standard Deviations)

		Mean (dB SPL)			Minimum (dB SPL)			Maximum (dB SPL)		
Group	Gender	T1 NHA	T2 NHA	T2 HA	T1 NHA	T2 NHA	T2 HA	T1 NHA	T2 NHA	T2 HA
FT users	Male	60 ± 3	60 ± 5	59 ± 5	26 ± 2	27 ± 4	27 ± 4	70 ± 4	71 ± 4	69 ± 3
	Female	60 ± 3	60 ± 4	59 ± 5	27 ± 2	26 ± 4	27 ± 4	69 ± 3	70 ± 5	67 ± 4
Experienced users	Male	60 ± 3	60 ± 4	59 ± 5	26 ± 2	26 ± 3	27 ± 3	67 ± 2	69 ± 4	68 ± 4
	Female	60 ± 3	60 ± 4	58 ± 5	26 ± 2	27 ± 3	27 ± 3	67 ± 3	68 ± 3	67 ± 4
Control group	Male	57 ± 3						68 ± 3		
	Female	59 ± 2						69 ± 3		

*Note.* SPL = sound pressure level; FT = first-time users; NHA = no hearing aid; HA = hearing aid.

### Between-Groups Measurements

#### f0

See Table 3 for an overview of all f0 values divided by gender, groups, sessions, and conditions. A one-way ANOVA was used to examine any differences in mean f0 between the FT users, experienced users, and the control group at baseline. There was a significant difference in mean f0 among the three male subgroups, *F*(2, 136) = 20.767, *p* < .001. Post hoc comparisons using the Tukey HSD test indicated that the mean f0 was significantly lower in the male control group (*m* = 126 ± 11 Hz) than in the male experienced users (149 ± 19 Hz). Likewise, there was a significant difference in mean f0 for the female subgroups, *F*(2, 98) = 25.593, *p* < .001, with post hoc tests indicating that the experienced female users had a significantly higher mean f0 (199 ± 15 Hz) than the females in the control group (175 ± 19 Hz). The results indicate that the difference in mean f0 was only significant between experienced users and the control group.

A one-way ANOVA indicated significant difference in minimum f0 between the male, but not female, groups at baseline, *F*(2, 136) = 37.397, *p* < .001. Tukey post hoc comparisons indicated that all three male subgroups differed significantly from each other (FT users 79 ± 10 Hz; experienced users 90 ± 14 Hz; Control group 70 ± 6 Hz). The results indicate that experienced users speak with a higher minimum f0 than the two other groups in the male subsample. Based on a one-way ANOVA, there were no significant differences in maximum f0 between any of the groups.

#### SPL

See Table 4 for an overview of the values for all SPL values divided by gender, groups, sessions, and conditions. A one-way ANOVA of mean SPL indicated significant differences in the male, but not the female, groups at baseline, *F*(2, 136) = 14.032, *p* < .001, for the males and *p* = .049 for the females). Tukey post hoc comparisons indicated that the recordings of the male FT users had a significantly higher mean SPL (59.6 ± 3 dB SPL) than the male control group participants (57 ± 3 dB SPL), *p* < .001. Additionally, the male experienced users also had a higher mean SPL (60 ± 3 dB SPL) than the male control group participants (57 ± 3 dB SPL), *p* < .001.

These results indicate that the mean produced SPL in males is higher with a hearing loss than without a hearing loss. The comparison was not significant for the females in the current study, although the control group had a lower mean SPL than the two other groups (see Table 4).

A one-way ANOVA indicated no significant differences for minimum SPL in either subsample at the beginning of hearing aid fitting (*p* > .005). One-way ANOVAs indicated significant differences in maximum SPL in both the male, *F*(2, 136) = 9.284, *p* < .001, and female, *F*(2, 98) = 5.892, *p* = .004, subsamples. Tukey post hoc comparisons for the male subsamples indicated that the FT users (70 ± 4 dB SPL) on average had a significantly higher maximum SPL in the recordings compared to the control group (68 ± 3 dB SPL), *p* = .003, and the experienced users (67 ± 3 dB SPL), *p* < .001. The comparison of male experienced users to the control group was not significant (*p* > .005). In the female subsample, the FT users did not significantly differ from the control group in terms of maximum SPL (*p* > .005). The only significant comparison was between the female FT users (70 ± 3 dB SPL) and the experienced users (67 ± 3 dB SPL), *p* = .002. The results indicate a possible effect of hearing loss on maximum SPL; however, the result of the analysis is inconsistent.

#### Perturbation Measurements

One-way ANOVAs at the beginning of hearing aid fitting were used to investigate any differences in the acoustic measurements of jitter, shimmer, and HNR. There were no significant differences in any of the tests, indicating that these measurements were on average relatively equal in each group and that a hearing loss did not significantly affect these measures.

### Acoustic Analysis: The Effect of Hearing Aids on Phonation During Recordings

A repeated measures mixed-model approach was used on data from the second session to predict the effect of acoustic values based on hearing condition, gender, inserts, and OVP as factors, with age as a continuous predictor.

#### f0

The results of mean f0 showed neither a significant main effect of hearing aids nor a significant main effect of groups (*p* > .05), but a significant interaction between Hearing Aid and OVP, *F*(1, 161) = 6.7, *p* = .011, η^2^= .04. The post hoc comparisons indicate that patients reporting OVP spoke with a higher mean f0 (men: *n* = 30, *m* = 142 Hz, *SE* = 2; women: *n* = 15, *m* = 201 Hz, *SE* = 4) than patients who did not report OVP (men: *n* = 76, *m* = 136 Hz, *SE* = 2; women: *n* = 49, *m* = 196 Hz, *SE* = 2).

There was also a significant effect of Hearing Aid × Gender × OVP, *F*(1, 161) = 4.9, *p* = .028, η^2^_p_ = .03. The post hoc analysis showed that the only significant difference in mean f0 was for the unaided recordings of women, where those reporting OVP had a slightly lower mean f0 (*n* = 49, *m* = 196 Hz, *SE* = 2) compared to those without OVP (*n* = 15, *m* = 205 Hz, *SE* = 1).

The between-subjects analysis showed a significant main effect of gender, *F*(1, 161) = 405.9, *p* < .001, η^2^= .72. The results showed that the women had a higher mean f0 (*n* = 64, *m* = 198 Hz, *SE* = 3) than the men (*M* = 106, *m* = 140 Hz, *SE* = 2). Age was also significant in the between-groups analysis, *F*(1, 61) = 13.3, *p* < .01, η^2^ =.076, indicating higher f0 with age. This is in line with previous studies of f0 changes in the elderly.

The same model was used to analyze the minimum f0. The result showed no significant main effect of hearing aids, nor any significant within-subjects interactions, *p* > .05. The between-subjects analysis identified significant main effects of Group and Gender. The post hoc analysis confirmed that the FT users had on average a lower minimum f0 (*n* = 85, *m* = 85 Hz, *SE* = 2) than the experienced users (*n* = 85, *m* = 91 Hz, *SE* = 2), *F*(1, 161) = 5.6, *p* = .019, η^2^= .034. The post hoc tests also showed a gender difference in f0, *F*(1, 161) = 48.4, *p* < .001, η^2^= .231. Men (*n* = 106, *m* = 81 Hz, *SE* = 1) had a lower minimum f0 on average than the women (*n* = 64, *m* = 96 Hz, *SE* = 2).

The result of maximum f0 showed no significant main effect of hearing aids, nor any significant within-subjects interactions, *p* > .05. The between-subjects analysis identified Gender as a significant factor, *F*(1, 161) = 6.8, *p* = .01, η^2 ^= .041. In this case, women had a higher maximum f0 (*n* = 64, *m* = 400 Hz, *SE* = 13) than the men (*n* = 106, *m* = 357 Hz, *SE* = 10).

#### SPL

The analysis of mean SPL found that hearing aids was a significant main effect for mean SPL, *F*(1, 161) = 9.3, *p* = .003, η^2 ^= .06. The speech recordings during the unaided condition were on average higher (*n* = 170, *m* = 60 dB SPL, *SE* = 3) than during the aided condition (*n* = 170, *m* = 58 dB SPL, *SE* = 4), a difference of 2 dB. There was a significant interaction between hearing aids and age, *F*(1, 161) = 4.7, *p* = .031, η^2 ^= .029, indicating that older participants spoke with a higher SPL when using hearing aids than younger. The between-subjects tests did not show any significant effects of the factors and interactions included in the model.

The minimum SPL showed no significant within-subjects main effect of hearing aids, nor any significant within-subjects interactions (*p* > .05). However, the OVP factor was significant in the between-subjects analysis, *F*(1, 161) = 7.4, *p* = .007, η^2 ^= .044 (No-OVP; *m* = 26 dB SPL, *SE* = 3 and OVP; *m* = 28 dB SPL, *SE* = 5), where users with OVP had 2 dB higher minimum SPL than users with No-OVP.

The interaction between OVP and Gender was also significant, *F*(1, 161) = 4.9, *p* = .029, η^2 ^= .03. In this case, the women with OVP had a higher minimum SPL (*m* = 29 dB SPL, *SE* = 1) compared to the women without OVP (*m* = 26 dB SPL, *SE* = 4), a difference of 3 dB. The difference in the male group was not significant.

The analysis of maximum SPL did not indicate a main effect of hearing aids, *p* > .05 while hearing aids and Gender (*p* < .001) was the only significant within-subjects interaction, *F*(1, 161) = 3.9, *p* = .048, η^2 ^= .024. The post hoc tests indicated that the women during the aided condition phonated with a lower maximum SPL (*n* = 64, *m* = 67 dB SPL, *SE* = 6) than in the unaided condition (*n* = 64, *m* = 70 dB SPL, *SE* = 6), a difference of 2 dB. For the men, the mean difference between the unaided (*n* = 106, *m* = 70 dB SPL, *SE* = 5) and aided (*n* = 106, *m* = 69 dB SPL, *SE* = 4) was smaller (1 dB) but still significant (*p* < .001). None of the between-subjects effects were significant.

#### Perturbation Measurements (Jitter, Shimmer, and HNR)

The result for jitter, shimmer, and HNR did not indicate any significant factors or interactions in either the within-subjects or between-subjects analysis.

## Discussion

In this study, we explored the subject of own-voice perception and phonation in hearing aid users. The aim of a hearing aid is to help hearing speech and other sounds, but it will inevitably alter users’ voice signals.

While predictions exist about these changes, researchers and clinicians need to better understand whether and how hearing aid users perceive them. Among our participants, 17.6% of FT users and 18.8% of experienced users reported moderate to severe OVP, less than a previous survey (Høydal, 2017), likely due to differing definitions and cut-offs. Subsequent sections will discuss the participants’ subjective voice ratings, followed by how perceptual changes in the sound signal may impact the acoustic qualities of the voice.

The results of this study are based on various statistical analyses. It is important to note that these analyses aim to identify causal links between acoustic parameters, hearing aid features, individual characteristics, and perceptual domains. However, even statistically significant results, may or may not have limited clinical relevance, and each finding needs to be evaluated accordingly.

### Voice Evaluation

The participants completed a short, subjective voice evaluation, rating their own voice (live and recorded), a familiar voice (recorded), and an unfamiliar voice (recorded), with and without the hearing aids on. Regression models indicate that hearing aids likely affect perception of different voice qualities.

Hearing aids increased the perceived pitch of all external voices (Table 2). Although hearing aids do not alter the fundamental frequency, pitch perception is influenced by the brightness of the sound, which is related to its high-frequency spectral content (Lau et al., 2021). As hearing aids provide high-frequency amplification to compensate for sloping hearing loss, the increased high-frequency content is perceived as an increase in pitch. Dynamic range compression amplifies sounds at lower SPL to make them more audible, while not amplifying or even attenuating sounds at a higher SPL to prevent discomfort or distortion. Hearing aids typically reduce amplification when users speaks, due to the higher SPL resulting from the proximity of the sound source to the microphone (Dillon, 2012; Patel & Panahi, 2020). Most hearing aid manufacturers include features in their fitting software to address own-voice complaints (Groth, 2014). Hearing specialists can also adjust settings, such as low-frequency amplification, to improve the users’ perception of their own voice. This could explain why hearing aids were a significant factor for pitch for all external voices but not for the user's live voice.

A contributing factor could be the presence of BC sounds (Pörschmann, 2000; Reinfeldt et al., 2010), which are believed to contribute to a lower perceived pitch or brightness in the own-voice live compared to on a recording (Hullar, 2009). However, while the effect of BC sounds on the own-voice perception is theoretically likely based on previous models (Stenfelt & Goode, 2005), its influence on perceived pitch has not been confirmed in a scientific setting. It should be noted that the evaluation of the live voice is different than for recorded voices as the evaluation is done simultaneously with the voice production. Such dual task could have affected the evaluation of the live voice. Gender was a significant factor for the user's own voice, which is presumably due to the average difference in f0 between men and women (Pépiot, 2014).

Hearing aids increased the perceived monotony in the user's live own voice, possibly caused by the dynamic range compression. The hearing aids amplify external voices, affecting the perception of formants. Given the initial playback setting of 60 dB SPL, it is likely that the user's own voice reached the hearing aid microphone at a higher level than the external voices. Regardless of whether the hearing aid signal processing includes own-voice detection, the amplification during the user's speech is probably less than for external voices due to the fast-acting compression.

Most hearing aid users have high-frequency hearing loss, so reduced amplification of their own voice results in diminished perception of high-frequency sounds, likely contributing to the perceived monotony in the user's live own voice. Additionally, a shift in the AC/BC balance (Stenfelt, 2011) due to a proportional decrease in BC sounds, may alter the perceived prosody due to changes in the spectral configuration of the user's voice.

Hearing aids were associated with the live own voice and the unfamiliar voice being perceived as less natural. We hypothesized that hearing aids would more strongly affect the perception of familiar voices, due to the users’ strong internal representation of the voice. However, the results show that the unfamiliar voice was more affected by the hearing condition in terms of naturalness. All the familiar voices were from professional voice users while the unfamiliar voices were retrieved from people with an “average” voice quality. The selection of familiar voices was also limited (four), and participants’ familiarity with these speakers may not reflect strong internal representation of their voices.

Hearing aid inserts influenced the ratings of naturalness in the live own voice and the familiar voice, although the effect was stronger for the former. Previous studies describe that occlusion is perceived by hearing aid users as making the own-voice sound unnatural, and/or like talking in a barrel, so that the finding that mold users rated their live own voice as less natural aligns with previous literature (Dillon, 2012; Laugesen et al., 2011). Although there is a belief that molds are only fitted for individuals with profound hearing impairments in modern care, and that these users would not be as sensitive to occlusion during speech, this study indicates otherwise. Hearing aid molds were a significant factor in the GEE model, with no significant difference in hearing thresholds between the participants reporting OVP and those without.

Our GEE models showed that hearing aids were associated with lower pleasantness for both the live and recorded own voice, especially affecting the live voice. We hypothesized that some users would perceive it as more pleasant and some as more unpleasant with hearing aids on. As the experienced users in this study had over 2 years’ experience with hearing aid use, they were expected to have acclimatized to the hearing aid more than the FT users. We therefore expected them to prefer or at least be neutral about their own voice to a larger extent than new users. Additionally, the clearer signal of the own voice generated by the hearing aid would be preferrable to some participants. However, overall, users in this study tended to rate their own voice as slightly less pleasant with their new hearing aids on.

Observing the data as a difference variable, there is near normal distribution. When categorized, 64 users found their voice less pleasant with hearing aids, 57 scored no change, and 47 indicated increased pleasantness. Among experienced users, 31.8% scored a positive change (more pleasant) with hearing aid use, compared to 23.5% of FT users. Conversely, 40% of FT users scored a negative change, compared to 35.3% of the experienced users. This indicates that around a third of the users did not perceive a difference with versus without hearing aids, and that numerous participants in both groups felt that the hearing aid improved the pleasantness of the own voice.

The negative change reported by 40% of FT users and 35.3% of experienced users in the live own voice may partly be due to the occlusion effect, as hearing aid molds were also associated with lower perceived pleasantness. This is in line with previous literature (Dillon, 2012), as users usually find the occlusion effect to be unpleasant, though occlusion is unlikely to affect external voices as it originates from BC sounds. The impact of hearing aids on both the live and recorded own voice indicates that occlusion is not the only cause. It is possible that the effect on the recorded own voice is a combination of sound processing and vocal implicit egotism (Hughes & Harrison, 2013), as pleasantness was not affected in the familiar voice. The familiar voice had (on average) the highest ratings of pleasantness, perhaps due to the voices being collected from professional voice users, with extensive experience in reading text out loud and perfecting their voice.

For the live own voice, the shift in AC/BC balance caused by hearing aids may influence the ratings. This is consistent with a previous survey (Høydal, 2017) where only 41% of users were satisfied with own-voice sounds and the sample consisted mostly of open-fitted patients.

General high-frequency amplification may also influence this. However, there is no objective measurement of gain during speech in this study, and as the dynamic range compression typically lowers amplification for the user's own speech, it is possible that the hearing aid users do not experience a significant amplification when speaking. Nonetheless, several users reported removing their hearing aids for public speaking or singing, as they would otherwise use a too loud or quiet voice.

The stronger effect on the live voice compared to the recorded own voice may be because most people are not used to hearing their own voice recorded, and often find it unfamiliar or unpleasant due to the lack of BC sounds, or spatial filtering effects. The recorded own voice does not match internal expectations, which can cause unease (Hullar, 2009).

To summarize the results from the voice evaluation, hearing aids seem to have a specific effect on the own voice during speech which differs from the sound processing of external voices. Familiarity seems to have little impact on perceived sound quality changes when using hearing aids, but implicit vocal egoism may be important. This suggests that the mechanism which causes OVP is triggered by a mechanism that mostly affects the internal representation of one's own voice.

The analysis of the voice evaluation scores showed that those with OVP rated the live own voice as more monotonous, less natural and pleasant during hearing aid use. The external voices were only rated as having a higher pitch during hearing aid use. This indicates that OVP is likely due to the sound processing of self-generated sounds, since the same group did not rate the recorded own voice in a similar manner.

These findings should be of interest to hearing clinicians and hearing aid developers to tailor hearing aids to provide a more realistic rendition of the users’ own voice as well as the voices of others. An understanding of how hearing aid users perceive voices can also be beneficial to speech therapists, as voice therapy typically relies heavily on auditory feedback.

### Acoustic Analysis

The results from the acoustic analysis indicate that hearing loss and hearing aids are likely to influence certain parameters in phonation, as auditory monitoring helps regulate our voice (Keough et al., 2013; Lane & Tranel, 1971). The role of the auditory system during phonation has been previously studied by altering the auditory environment and observing vocal response (Lane & Tranel, 1971; Purcell & Munhall, 2006a, 2006b; Villacorta et al., 2007; Xu et al., 2004). However, these studies were conducted in controlled environments and relatively little is known about how sensory changes from hearing impairment or hearing aid use affect vocal regulation. While other studies have examined acoustic parameters in people with hearing impairment, most of the previous research has been conducted on deaf individuals or individuals with profound hearing loss, often acquired in childhood (Coelho, Medved, et al., 2015). Since elderly individuals make up the biggest category of people with hearing loss, and as this is generally treated with hearing aids, further studies are needed.

Due to methodological reasons, both groups with hearing impairment were recorded only under the unaided condition at baseline (the FT users had not received their hearing aid yet and many of the experienced users had broken or lost theirs). Below is a discussion regarding key findings from the baseline analysis and the mixed-model comparisons from the second visit.

#### Fundamental Frequency

The baseline comparisons between-groups and the mixed-model analysis showed an interesting, but unclear, relationship between hearing impairment, hearing aids, and f0.

At baseline, both hearing-impaired groups had a higher mean f0 than the control group for both genders, but statistically, only the male experienced group had a significantly higher mean f0 than the males in the control group and male FT users.

In the current study, the mean difference between the hearing-impaired groups and the control group was +23 Hz. Despite methodological differences such as sample size, age, and recording procedures the results appear to be in line with the two previous studies that found that f0 in elderly women with hearing loss increased with sloping audiometric curves (Baraldi Gdos et al., 2007), and approximately 17 Hz higher f0 in those with hearing impairment than those without (Mora et al., 2012).

The experienced users were on average 5 years older than the FT users and the control group. Studies show that f0 varies across the life span. Men's voices tend to increase in f0 after 40 years of age, while women's f0 tends to decrease post-menopause and rise again around 70 years of age (Baken, 2005). However, findings regarding age-related changes in f0 are inconsistent (Nishio & Niimi, 2008). It is unclear whether the observed difference in f0 in the current study can reasonably be attributed to an age difference. There was no significant correlation between age and f0, possibly due to the limited inclusion of younger or middle-aged participants. We therefore conclude that age differences offer at most a partial explanation for these results.

In the mixed-model follow-up analysis, hearing aids were not a significant main effect in predicting mean f0. The interactions “hearing aids × OVP” and “hearing aids × gender × OVP” were, however, significant. Participants reporting OVP phonated with a higher mean f0 than those without OVP, with significant post hoc results for female participants. The results are insufficient for a satisfactory explanation and the authors think the subject needs to be explored using more direct technological approaches to evaluate what is happening in the hearing aids and in the user's ear.

The analysis of minimum f0 revealed groupwise differences at baseline, with hearing-impaired participants showing significantly higher minimum f0 than the control group for the male subsample. The repeated measures mixed-model ANOVA found a significant main effect of Group × Gender, where minimum f0 was significantly lower for FT users compared to experienced users, but no significant effect of hearing aids.

Overall, the findings suggest that hearing impairment is linked to an increase in mean f0. Hearing aids generally seem to have no effect on f0 for most users, but they may influence f0 for those experiencing OVP. These findings should be of interest to those working with voice therapy, as changes in f0 can strain the vocal folds.

#### Speech SPL

The baseline results show that the unaided participants with hearing impairment on average spoke with approximately 1–3 dB higher levels than the control group, with a significant difference only among men, possibly due to fewer female participants in the sample. A 2013 study found that older adults tend to speak with lower SPL than younger individuals. The study excluded hearing aid users and individuals whose hearing was not considered age-adequate (Goy et al., 2013). In this study, the youngest group (the control group) spoke more softly.

Since the term “the Lombard effect” was coined, many studies have examined under which conditions it occurs, and which parameters are affected. In relation to hearing loss, the individual's perception of SPL has decreased, and generally also the ability to discriminate sounds. This study indicates that people with hearing impairment will increase speech levels to match previous internal representations of their own voice. The difference in SPL was relatively small. This indicates that hearing aid users may rely more on proprioception to achieve an appropriate voice level.

The mixed-model analysis of unaided/aided measures after hearing aid fitting showed a main effect of hearing aids on average SPL. Hearing aids decreased SPL by 2 dB, which is likely due to the amplification in the devices restoring auditory perception to better fit the internal expectations of voice SPL, consistent with previous research (Stenfelt, 2012). In the aforementioned study, 30 participants phonated with a lower SPL when using hearing aids, exhibiting a 2–4 dB difference.

The results from the baseline comparisons and the mixed-model analysis both point to a Lombard-like effect, where decreased feedback from hearing impairment increases produced SPL, while feedback gain from amplification decreases it.

Previous studies by the authors examined scores from the standardized form Voice Handicap Index and found that the most commonly reported voice problems fall under the “functional” category (Hengen et al., 2018, 2020). Examples include statements like “My voice makes it difficult for people to hear me,” “Other people have a hard time understanding me in a noisy environment,” “My family has trouble hearing me when I call for them in another part of the house.” These statements can reflect problems arising from different types of voice problems, but considering the results from the acoustic analysis as well as those from Laugesen et al. (2011) it can be argued that sound pressure regulation is a probable explanation for the scores in this study. These findings are likely useful when diagnosing and treating voice problems in hearing aid users clinically to identify the root cause of the complaints.

Hearing aids did not significantly affect minimum or maximum SPL, although the results indicated potential interaction effects between OVP, gender, and hearing aids. Women appear to have a larger reduction (−2 dB) in maximum SPL than men (−1 dB) when using hearing aids. Unaided baseline comparisons showed that experienced users had a significantly lower maximum SPL compared to the two other groups. This finding may indicate that greater hearing loss leads to a more restricted SPL range, which can be attributed to the reduced dynamic range typically associated with higher degrees of hearing loss.

The SPL values in this study are low compared to a previous study of normative voice values in younger and older adults (Goy et al., 2013), likely due to differences in recording procedures. The study by Goy et al. (2013) used a microphone positioned 1.5 cm from the participants’ lips; the current study used a microphone approximately 20–30 cm away.

#### Perturbation Measures

Overall, the perturbation measures collected in this study (jitter, shimmer, HNR) did not appear to be influenced by hearing impairment or hearing aids, suggesting that hearing aids likely do not affect harmonious aspects of the voice in the short term. Baseline comparisons indicate that neither hearing impairment nor hearing aid use directly affect disharmonic voice quality. This conforms with findings from one of our previous studies (Hengen et al., 2018) and the participants’ own-voice evaluation, which showed no perceived increase in hoarseness during hearing aid use. Intriguingly, while the perturbation measures were normal and equal at baseline, there was a notable difference in perceived voice problems between the groups. It is important to note that voice problems encompass more than just hoarseness. Additionally, despite their common use in clinic and research settings, the perturbation measures in this study have faced criticism for their reliability and validity. A 2018 study showed that there were no significant differences in jitter or shimmer between a group of patients with voice disorders compared to a control group. They conclude, however, that the measurements can be improved when instructing patients to speak at a certain level (Brockmann-Bauser et al., 2018).

### Study Limitations

This study employed various methods to understand the perceptual and functional effects that hearing impairment and hearing aids may have on the voice, each with its limitations.

Own-voice perception is inherently subjective, and subjective ratings introduce methodological challenges. In our study, variability in ratings may arise from the questionnaire design or participants’ difficulty evaluating voices. Using an unverified, self-developed evaluation form complicates the assessment of variability due to confounding factors. Average intra-rater variability is as an example not known.

Allowing participants to choose re-listen to the recordings introduces a slight confounding factor. However, this was considered necessary, as perceptual voice evaluation was likely a novel and difficult task for most participants. Allowing repeats of recordings was thought to improve their judgment of the parameters in question, a common practice in professional voice evaluations.

The current study did not compare the voice evaluations across different sessions, as it was believed that the results would be too affected by intra-rater variability to be considered meaningful. The results of the repeated mixed-model analysis are based on evaluations conducted within 20 min of each other, which we believe minimizes variability due to mood changes or other confounding factors.

The voice evaluations and recordings which the acoustic analysis is based on were conducted in a sound-attenuated booth. This is standard procedure to avoid interference in the recordings. However, this controlled environment may affect the hearing aids’ sound processing. As noted earlier in the discussion, a quiet setting means that the hearing aid does not have to adjust for multiple speakers or noise reduction in the same way it operates in everyday environments.

Another limitation is that many experienced hearing aid users participated due to refitting after losing or having malfunctioning devices, which is a common reason for seeking refitting. Excluding these participants would have significantly reduced the sample size. However, these users had an average of 13 years of hearing aid experience prior to loosing or damaging their devices. A smaller comparison study involving experienced hearing aid users with normally functioning hearing aids would be valuable.

Modifying the auditory environment during recordings or voice evaluations could yield different results and should be considered in future studies, but the incompatibility of hearing aids with headphones complicates this. A surround effect may be achieved using multiple loudspeakers, but this option was not available to the authors in the current study.

This study used many statistical tests to analyze the results, increasing the chance of false positives. An adjusted *p*-value was used to mitigate this risk, but there is no consensus for which adjustment method is the most appropriate (Perneger, 1998). In this study, the calculated *p*-value is based on the number of comparisons for the same parameter.

## Conclusions

Hearing aids appeared to increase the user's perceived pitch of external voices while increasing the likelihood of higher ratings for monotony and lower pleasantness in their own voice during speech. The use of molds was associated with lower ratings of naturalness and pleasantness in their own live voice. Participants reporting OVP rated a larger increase in monotony, lower naturalness and pleasantness in their own live voice, compared to participants without OVP. Consequently, OVP in the current sample seems to be triggered primarily by mechanisms that affect internal representation of one's own voice.

Acoustic analysis found that hearing aid use on average resulted in an immediate adaptation of SPL, but not f0 or perturbation values at large. There were differences between the groups of participants with hearing impairment and the control group which suggest that there might be a long-term effect of hearing aid use on certain aspects of f0 and SPL during speech. The female group with OVP had a higher mean f0, but other acoustic measures did not suggest a significant difference to those not experiencing own-voice issues.

This study confirms previous literature regarding the interrelationship between hearing and the voice. The results should motivate clinicians to routinely consider hearing functions when assessing voice problems.

## Supplemental Material

sj-docx-1-tia-10.1177_23312165251322064 - Supplemental material for Effect of Hearing Aids on Phonation and Perceived Voice Qualities Supplemental material, sj-docx-1-tia-10.1177_23312165251322064 for Effect of Hearing Aids on Phonation and Perceived Voice Qualities by Johanna Hengen, Inger Lundeborg Hammarström and Stefan Stenfelt in Trends in Hearing
