# Supplementary material for: Effect of Hearing Aids on Phonation and Perceived Voice Qualities
Source: Trends Hear. 2025 Mar 3;29:23312165251322064. doi: 10.1177/23312165251322064 (PMC11873921; doi:10.1177/23312165251322064)
Supplement: sj-docx-1-tia-10.1177_23312165251322064 - Supplemental material for Effect of Hearing Aids on Phonation and Perceived Voice Qualities [file sj-docx-1-tia-10.1177_23312165251322064.docx]

Attachment 1. Average ratings of voice qualities of the own (live) voice and the own (recorded) voice for all groups and sessions.

1) Pitch ratings.

|  |  |  | Live own voice | | | Recorded own voice | | |
| --- | --- | --- | --- | --- | --- | --- | --- | --- |
| Group | Gender | N | T1 Unaided | T2  Unaided | T2  Aided | T1 Unaided | T2  Unaided | T2  Aided |
| FT-users | Male | 54 | 3.6 ±1.2 | 3.4 ±1.2 | 3.9 ±1.0 | 3.9 ±1.6 | 3.5 ±1.0 | 3.8 ±1.2 |
|  | Female | 31 | 3.9 ±1.3 | 3.9 ±1.1 | 4.7 ±1.8 | 4.3 ±1.6 | 3.7 ±1.4 | 4.2 ±1.6 |
| Exp Users | Male | 52 | 3.4 ±1.3 | 3.5 ±1.4 | 3.5 ±1.3 | 3.2 ±1.2 | 3.2 ±1.1 | 3.5 ±1.4 |
|  | Female | 33 | 3.9 ±1.3 | 4.0 ±1.2 | 3.7 ±1.3 | 3.9 ±1.3 | 3.8 ±1.2 | 4.1 ±1.3 |
| Control group | Male | 33 | 3.6 ±1.2 | N/a | N/a | 3.5 ±1.4 | N/a | N/a |
|  | Female | 37 | 3.7 ±1.1 |  |  | 3.7 ±1.2 |  |  |
|  |  |  | Unfamiliar voice | | | Familiar voice | | |
| Group | Gender | N | T1 Unaided | T2  Unaided | T2  Aided | T1 Unaided | T2  Unaided | T2  Aided |
| FT-users | Male | 54 | 4.3 ±1.1 | 4.0 ±1.1 | 4.3 ±1.0 | 3.4 ±1.1 | 3.7 ±1.0 | 4.0 ±1.1 |
|  | Female | 31 | 4.8 ±1.2 | 4.5 ±1.4 | 4.8 ±1.3 | 3.8 ±1.3 | 3.8 ±1.2 | 4.3 ±1.5 |
| Exp Users | Male | 52 | 4.2 ±1.2 | 4.1 ±1.4 | 4.5 ±1.1 | 3.6 ±1.5 | 3.8 ±1.4 | 4.1 ±1.4 |
|  | Female | 33 | 4.4 ±1.4 | 4.0 ±1.3 | 4.6 ±1.3 | 3.7 ±1.5 | 4.1 ±1.4 | 4.2 ±1.1 |
| Control group | Male | 33 | 4.2 ±1.2 | N/a | N/a | 3.3 ±1.4 | N/a | N/a |
|  | Female | 37 | 3.9 ±1.3 |  |  | 3.4 ±1.1 |  |  |

2) Strain

|  |  |  | Live own voice | | | Recorded own voice | | |
| --- | --- | --- | --- | --- | --- | --- | --- | --- |
| Group | Gender | N | T1 Unaided | T2  Unaided | T2  Aided | T1 Unaided | T2  Unaided | T2  Aided |
| FT-users | Male | 54 | 3.6 ±1.6 | 3.8 ±1.7 | 3.8 ±1.7 | 4.2 ±1.4 | 4.2 ±1.5 | 3.7 ±1.5 |
|  | Female | 31 | 3.3 ±1.4 | 3.3 ±1.6 | 3.7 ±1.9 | 3.1 ±1.5 | 3.2 ±1.6 | 3.2 ±1.8 |
| Exp Users | Male | 52 | 3.5 ±1.5 | 3.7 ±1.5 | 3.5 ±1.5 | 3.3 ±1.6 | 3.4 ±1.6 | 3.3 ±1.7 |
|  | Female | 33 | 3.9 ±1.7 | 3.9 ±1.6 | 3.8 ±1.5 | 3.6 ±1.8 | 3.5 ±1.9 | 3.5 ±1.6 |
| Control group | Male | 33 | 3.5 ±3.5 | N/a | N/a | 3.7 ±1.6 | N/a | N/a |
|  | Female | 37 | 3.6 ±3.9 |  |  | 3.1 ±1.7 |  |  |
|  |  |  | Unfamiliar voice | | | Familiar voice | | |
| Group | Gender | N | T1 Unaided | T2  Unaided | T2  Aided | T1 Unaided | T2  Unaided | T2  Aided |
| FT-users | Male | 54 | 3.7 ±1.7 | 3.6 ±1.6 | 3.7 ±1.4 | 1.6 ±1.8 | 1.9 ±1.5 | 1.7 ±1.6 |
|  | Female | 31 | 3.8 ±1.9 | 3.7 ±1.6 | 3.7 ±1.7 | 1.5 ±1.8 | 1.7 ±1.7 | 1.9 ±1.8 |
| Exp Users | Male | 52 | 3.4 ±1.7 | 3.2 ±1.7 | 3.5 ±1.5 | 1.9 ±1.7 | 1.7 ±1.6 | 1.4 ±1.4 |
|  | Female | 33 | 3.9 ±1.5 | 4.1 ±1.6 | 3.8 ±1.7 | 2.3 ±1.6 | 2.5 ±1.5 | 2.2 ±1.8 |
| Control group | Male | 33 | 3.5 ±1.8 | N/a | N/a | 1.6 ±1.5 | N/a | N/a |
|  | Female | 37 | 4.0 ±1.8 |  |  | 1.3 ±1.7 |  |  |

3). Hoarseness ratings.

|  |  |  | Live own voice | | | Recorded own voice | | |
| --- | --- | --- | --- | --- | --- | --- | --- | --- |
| Group | Gender | N | T1 Unaided | T2  Unaided | T2  Aided | T1 Unaided | T2  Unaided | T2  Aided |
| FT-users | Male | 54 | 3.7 ±1.8 | 3.9 ±1.6 | 3.6 ±1.6 | 4.0 ±1.8 | 3.9 ±1.7 | 3.9 ±1.7 |
|  | Female | 31 | 3.0 ±1.8 | 3.1 ±1.8 | 3.5 ±1.6 | 3.7 ±1.8 | 3.1 ±1.8 | 3.6 ±2.1 |
| Exp Users | Male | 52 | 3.9 ±1.9 | 3.9 ±1.7 | 3.8 ±1.9 | 3.5 ±2.0 | 3.4 ±1.9 | 3.7 ±1.9 |
|  | Female | 33 | 4.1 ±1.6 | 3.8 ±1.7 | 3.9 ±1.6 | 3.2 ±1.7 | 3.6 ±1.8 | 3.7 ±1.8 |
| Control group | Male | 33 | 3.3 ±1.9 | N/a | N/a | 3.0 ±1.8 | N/a | N/a |
|  | Female | 37 | 2.9 ±1.9 |  |  | 2.7 ±2.1 |  |  |
|  |  |  | Unfamiliar voice | | | Familiar voice | | |
| Group | Gender | N | T1 Unaided | T2  Unaided | T2  Aided | T1 Unaided | T2  Unaided | T2  Aided |
| FT-users | Male | 54 | 2.6 ±1.6 | 3.1 ±1.8 | 3.2 ±1.5 | 1.7 ±1.8 | 2.2 ±1.8 | 1.8 ±1.7 |
|  | Female | 31 | 2.6 ±2.0 | 2.9 ±1.6 | 2.8 ±1.9 | 1.8 ±1.6 | 1.7 ±1.7 | 1.6 ±1.6 |
| Exp Users | Male | 52 | 3.0 ±1.8 | 3.0 ±1.7 | 2.9 ±1.7 | 1.7 ±1.7 | 1.8 ±1.7 | 1.5 ±1.5 |
|  | Female | 33 | 3.5 ±1.7 | 3.2 ±2.0 | 3.6 ±1.5 | 2.2 ±2.0 | 2.6 ±1.8 | 2.0 ±1.8 |
| Control group | Male | 33 | 2.5 ±1.8 | N/a | N/a | 1.2 ±1.5 | N/a | N/a |
|  | Female | 37 | 2.4 ±2.1 |  |  | 1.6 ±1.6 |  |  |

4). Prosody/Monotony ratings.

|  |  |  | Live own voice | | | Recorded own voice | | |
| --- | --- | --- | --- | --- | --- | --- | --- | --- |
| Group | Gender | N | T1 Unaided | T2  Unaided | T2  Aided | T1 Unaided | T2  Unaided | T2  Aided |
| FT-users | Male | 54 | 4.1 ±1.2 | 4.2 ±1.1 | 4.3 ±1.2 | 4.1 ±1.3 | 3.7 ±1.2 | 3.9 ±1.0 |
|  | Female | 31 | 3.5 ±1.1 | 3.8 ±1.3 | 4.4 ±1.2 | 3.9 ±1.2 | 3.7 ±1.2 | 3.7 ±1.0 |
| Exp Users | Male | 52 | 4.1 ±1.4 | 4.0 ±1.2 | 4.3 ±1.3 | 4.1 ±1.2 | 3.9 ±1.4 | 3.9 ±1.2 |
|  | Female | 33 | 3.6 ±1.2 | 4.0 ±1.3 | 4.1 ±1.4 | 3.7 ±1.2 | 3.8 ±1.1 | 3.8 ±1.3 |
| Control group | Male | 33 | 3.5 ±1.3 | N/a | N/a | 3.7 ±1.5 | N/a | N/a |
|  | Female | 37 | 3.6 ±1.4 |  |  | 3.3 ±1.3 |  |  |
|  |  |  | Unfamiliar voice | | | Familiar voice | | |
| Group | Gender | N | T1 Unaided | T2  Unaided | T2  Aided | T1 Unaided | T2  Unaided | T2  Aided |
| FT-users | Male | 54 | 4.3 ±1.1 | 4.0 ±1.1 | 4.3 ±1.0 | 2.9 ±1.1 | 2.9 ±1.1 | 2.9 ±1.2 |
|  | Female | 31 | 4.8 ±1.2 | 4.5 ±1.4 | 4.8 ±1.3 | 2.8 ±1.3 | 3.0 ±1.1 | 2.6 ±1.3 |
| Exp Users | Male | 52 | 4.2 ±1.2 | 4.1 ±1.4 | 4.5 ±1.1 | 2.7 ±1.2 | 2.9 ±1.5 | 2.9 ±1.3 |
|  | Female | 33 | 4.4 ±1.4 | 4.0 ±1.3 | 4.6 ±1.3 | 2.8 ±1.2 | 3.1 ±1.2 | 2.8 ±1.0 |
| Control group | Male | 33 | 4.2 ±1.2 | N/a | N/a | 2.7 ±1.2 | N/a | N/a |
|  | Female | 37 | 3.9 ±1.3 |  |  | 2.1 ±1.1 |  |  |

5). Naturalness ratings.

|  |  |  | Live own voice | | | Recorded own voice | | |
| --- | --- | --- | --- | --- | --- | --- | --- | --- |
| Group | Gender | N | T1 Unaided | T2  Unaided | T2  Aided | T1 Unaided | T2  Unaided | T2  Aided |
| FT-users | Male | 54 | 3.2 ±1.4 | 3.1 ±1.4 | 3.8 ±1.4 | 3.3 ±1.4 | 3.2 ±1.4 | 3.5 ±1.3 |
|  | Female | 31 | 2.6 ±1.7 | 2.8 ±1.7 | 3.2 ±1.5 | 3.1 ±1.6 | 2.9 ±1.6 | 3.4 ±1.8 |
| Exp Users | Male | 52 | 3.3 ±1.4 | 3.4 ±1.3 | 3.2 ±1.7 | 3.2 ±1.4 | 3.1 ±1.3 | 3.3 ±1.5 |
|  | Female | 33 | 3.2 ±1.3 | 3.3 ±1.3 | 3.2 ±1.8 | 3.6 ±1.3 | 3.3 ±1.3 | 3.1 ±1.6 |
| Control group | Male | 33 | 2.7 ±1.4 | N/a | N/a | 3.5 ±1.7 | N/a | N/a |
|  | Female | 37 | 2.7 ±1.7 |  |  | 2.8 ±1.6 |  |  |
|  |  |  | Unfamiliar voice | | | Familiar voice | | |
| Group | Gender | N | T1 Unaided | T2  Unaided | T2  Aided | T1 Unaided | T2  Unaided | T2  Aided |
| FT-users | Male | 54 | 3.0 ±1.3 | 3.2 ±1.3 | 3.4 ±1.4 | 1.8 ±1.5 | 1.9 ±1.4 | 1.9 ±1.4 |
|  | Female | 31 | 3.5 ±1.3 | 2.9 ±1.3 | 3.0 ±1.5 | 1.5 ±1.7 | 2.2 ±1.9 | 2.2 ±1.9 |
| Exp Users | Male | 52 | 2.9 ±1.5 | 2.6 ±1.0 | 2.9 ±1.6 | 1.7 ±1.5 | 1.9 ±1.4 | 1.9 ±1.4 |
|  | Female | 33 | 3.3 ±1.5 | 3.4 ±1.5 | 3.4 ±1.4 | 2.1 ±1.7 | 2.0 ±1.5 | 2.0 ±1.6 |
| Control group | Male | 33 | 3.0 ±1.5 | N/a | N/a | 1.6 ±1.6 | N/a | N/a |
|  | Female | 37 | 3.0 ±1.9 |  |  | 1.2 ±1.5 |  |  |

6). Pleasantness ratings.

|  |  |  | Live own voice | | | Recorded own voice | | |
| --- | --- | --- | --- | --- | --- | --- | --- | --- |
| Group | Gender | N | T1 Unaided | T2  Unaided | T2  Aided | T1 Unaided | T2  Unaided | T2  Aided |
| FT-users | Male | 54 | 3.6 ±1.3 | 3.6 ±0.9 | 3.8 ±1.2 | 3.8 ±1.0 | 3.5 ±1.1 | 3.7 ±1.0 |
|  | Female | 31 | 3.1 ±0.9 | 3.0 ±1.0 | 3.4 ±1.0 | 3.7 ±1.3 | 3.1 ±1.0 | 3.7 ±1.3 |
| Exp Users | Male | 52 | 3.4 ±1.1 | 3.5 ±1.2 | 3.6 ±1.2 | 3.6 ±1.0 | 3.3 ±1.1 | 3.4 ±1.1 |
|  | Female | 33 | 3.5 ±1.1 | 3.5 ±1.4 | 4.0 ±1.3 | 3.7 ±1.4 | 3.7 ±1.3 | 3.6 ±1.1 |
| Control group | Male | 33 | 3.3 ±1.2 | N/a | N/a | 3.7 ±1.2 | N/a | N/a |
|  | Female | 37 | 3.1 ±1.4 |  |  | 3.5 ±1.5 |  |  |
|  |  |  | Unfamiliar voice | | | Familiar voice | | |
| Group | Gender | N | T1 Unaided | T2  Unaided | T2  Aided | T1 Unaided | T2  Unaided | T2  Aided |
| FT-users | Male | 54 | 3.3 ±1.2 | 3.3 ±1.2 | 3.5 ±1.1 | 1.8 ±0.9 | 1.9 ±1.0 | 2.1 ±1.0 |
|  | Female | 31 | 3.3 ±1.2 | 3.3 ±1.3 | 3.3 ±1.4 | 1.9 ±0.9 | 1.8 ±1.0 | 2.3 ±1.4 |
| Exp Users | Male | 52 | 3.3 ±1.1 | 3.1 ±1.0 | 3.3 ±1.1 | 2.0 ±0.7 | 2.1 ±1.0 | 1.8 ±1.0 |
|  | Female | 33 | 3.6 ±1.2 | 3.8 ±1.1 | 3.9 ±1.2 | 2.0 ±0.9 | 2.5 ±1.3 | 2.0 ±1.0 |
| Control group | Male | 33 | 3.4 ±1.3 | N/a | N/a | 1.7 ±0.8 | N/a | N/a |
|  | Female | 37 | 3.4 ±1.3 |  |  | 1.6 ±1.2 |  |  |
